# Oral glucocorticoid therapy and all-cause and cause-specific mortality in patients with rheumatoid arthritis: a retrospective cohort study

**DOI:** 10.1007/s10654-016-0167-1

**Published:** 2016-06-02

**Authors:** Mohammad Movahedi, Ruth Costello, Mark Lunt, Stephen Richard Pye, Jamie Christopher Sergeant, William Gregory Dixon

**Affiliations:** 1 Arthritis Research UK Centre for Epidemiology, Centre for Musculoskeletal Research, Institute for Inflammation and Repair, Manchester Academic Health Science Centre, The University of Manchester, Manchester, UK; 2 NIHR Manchester Musculoskeletal Biomedical Research Unit, Central Manchester University Hospitals NHS Foundation Trust, Manchester Academic Health Science Centre, Manchester, UK; 3 Health eResearch Centre, Manchester Academic Health Science Centre, The University of Manchester, Manchester, UK

**Keywords:** Rheumatoid arthritis, Glucocorticoids, Mortality, Steroids

## Abstract

Previous studies of glucocorticoid (GC) therapy and mortality have had inconsistent results and have not considered possible perimortal bias—a type of protopathic bias where illness in the latter stages of life influences GC exposure, and might affect the observed relationship between GC use and death. This study aimed to investigate all-cause and cause-specific mortality in association with GC therapy in patients with rheumatoid arthritis (RA), and explore possible perimortal bias. A retrospective cohort study using the primary care electronic medical records. Oral GC exposure was identified from prescriptions. Mortality data were obtained from the UK Office for National Statistics. Multivariable Cox proportional hazards regression models assessed the association between GC use models and death. Several methods to explore perimortal bias were examined. The cohort included 16,762 patients. For ever GC use there was an adjusted hazard ratio for all-cause mortality of 1.97 (95 % CI 1.81–2.15). Current GC dose of below 5 mg per day (prednisolone equivalent dose) was not associated with an increased risk of death, but a dose–response association was seen for higher dose categories. The association between ever GC use and all-cause mortality was partly explained by perimortal bias. GC therapy was associated with an increased risk of mortality for all specific causes considered, albeit to a lesser extent for cardiovascular causes. GC use was associated with an increased risk of death in RA, at least partially explained by perimortal bias. Importantly, GC doses below 5 mg were not associated with an increased risk of death.

**Electronic supplementary material:**

The online version of this article (doi:10.1007/s10654-016-0167-1) contains supplementary material, which is available to authorized users.

## Introduction

Rheumatoid arthritis (RA) is a chronic inflammatory disease which affects between 0.5 and 1 % of the adult population worldwide [1–3]. Oral glucocorticoid **(**GC) therapy was introduced as a treatment for patients with RA nearly 60 years ago [4] and is still used widely. Around one third of patients with RA are current users, and two thirds of patients have ever used GCs [5]. GCs improve symptoms of active RA through reducing joint pain, swelling and stiffness [6]. However, there are some concerns about their potential side effects including cardiovascular (CV) events, diabetes, infection, fracture, and cataracts [7–11], many of which are associated with an increased risk of mortality.

Previous studies have investigated the association between GC therapy and mortality, mostly focusing on all-cause mortality, though some have investigated CV mortality [5, 12–16]. Findings from these studies are not consistent. GCs have been associated with an increased risk of all-cause mortality in some [12–15, 17, 18] but not all studies [16, 19, 20], with similar inconsistency for CV mortality [12, 14, 16]. Very few studies have examined other cause-specific mortality. In studies that consider dose, some have suggested no association with doses <5 mg prednisolone equivalent [12, 13], reflecting either a lack of significant side effects at this dose or perhaps a favourable balance between side effects and positive anti-inflammatory properties.

There are important methodological issues when considering GC exposure and mortality, including confounding by indication—whereby GC therapy is given to patients with high disease severity and high disease severity is itself associated with increased mortality. However, studies have rarely considered a form of protopathic bias we will call ‘perimortal bias’, where illness in the latter stages of life influences GC exposure, and which consequently might affect the observed relationship between GC use and death. For example, if a patient were to develop cancer, GC therapy may be prescribed to treat the malignancy and a resultant association would be observed between GCs and (cancer-specific) mortality. The aim of this study was to investigate all-cause and cause-specific mortality in association with various models of oral GC exposure in patients with RA, and to explore and control for the possible existence of perimortal bias.

## Methods

### Database

The Clinical Practice Research Datalink (CPRD) is a database of anonymised UK primary care electronic medical records covering 9 % of the population. There are 650 General Practitioner (GP) practices who contribute high-quality data, with over 5.5 million active patients who are broadly representative of the UK population [21, 22]. Information on the database includes patient demographics, medical diagnoses, clinical tests, hospital referrals, and drug prescriptions. Diagnoses on CPRD have been shown to have a high validity [23]. Selected practices consent to linkage to mortality data for England and Wales from the UK Office for National Statistics (ONS), and to Hospital Episode Statistics (HES), which provides information on hospital admissions.

### Study population

Patients with RA were identified in the CPRD database using a validated algorithm [24]. To satisfy the algorithm patients needed either: more than one RA Read code, a seropositive/erosive RA or “rheumatoid arthritis” code (such as RA of knee), and no code for an alternative diagnosis after the last RA code; or a DMARD prescription with no Read code for an alternative indication in the 5 years prior to the first DMARD prescription. A study window of 1st January 1998–1st October 2011 was used. The cohort was restricted to the 340 GP practices eligible to be linked to ONS mortality and HES data, with data restricted to the period of mortality data linkage for each GP practice to ensure accurate vital status information. Patients entered the study on the latest of first RA code, date of ONS linkage or 01/01/1998. Patients under 16 years of age were excluded. The population was also restricted to patients with at least 1 year’s information in CPRD prior to cohort entry, to allow assessment of prior GC exposure. Follow-up ended at transfer out of GP practice, GP practice data last collection, death, or 01/10/2011, whichever came first.

### Exposure definition

**Fig. 1:**
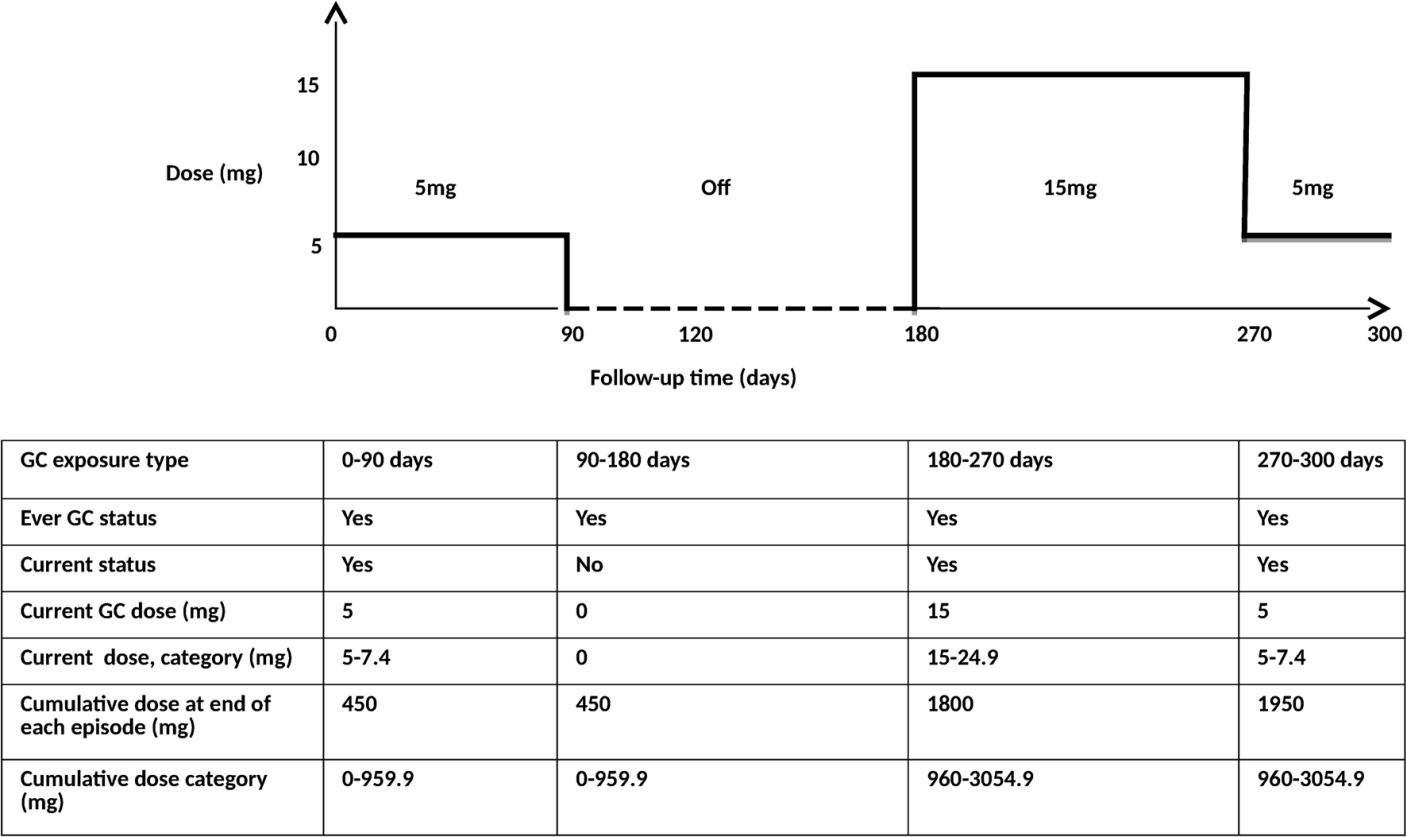
Example of GC exposure definitions during follow-up for a hypothetical patient

The dose and duration of each GC prescription was derived from the available prescription information using a pre-specified algorithm (see Online Resource item A1). Doses of oral GCs were converted into a prednisolone-equivalent dosage (PED). Time-varying GC exposure was then defined in six ways: (1) ever use: a patient was considered a never user until the point of their first GC prescription when they became an ever user. This was the primary analyses. (2) Current use: a patient was considered a current user during their GC prescription and became a non-user during the periods without a GC prescription. (3) Current dose (5 mg/day): during a patient’s GC prescription this was the dose divided by 5, during non-use this was zero. (4) Current dose category: a patient’s current dose was categorised into the following categories: non-use, >0–4.9, 5–7.4, 7.5–14.9, 15–24.9 and 25 mg and over PED/day. (5) Cumulative dose since cohort entry (1000 mg/day): a patient’s cumulative dose was calculated by summing the doses that had been prescribed up to that point and dividing by 1000, during non-use the cumulative dose would remain at the cumulative dose of prescriptions up to that point. (6) Cumulative dose category: a patent’s cumulative dose was categorised into the following categories: non-use, >0–959, 960–3054, 3055–7299 and 7300 mg and over PED/day. An example of a patient’s changing GC status through time is shown in Fig. 1. As time in hospital creates a gap in primary care records, because patients cannot attend the primary care practice, the GC exposure was set to the latest GC status prior to admission for the duration of any hospital inpatient stay identified using HES data.

### Death ascertainment

The ONS defines cause of death by International Classification of Diseases version 2010 (ICD 10) codes with a specified underlying cause of death. We examined the underlying cause of death by the most frequent ICD 10 chapter headings of circulatory (ICD chapter I), neoplasms (ICD chapters C and D), respiratory diseases (ICD chapter J), and the remaining chapter headings grouped together in an “other causes” category. We also identified the leading causes of death in each chapter. Causes of death prior to 2001 were coded using ICD 9 and were later mapped to ICD 10.

### Confounders

The following a priori potential confounders were included in the analyses: gender, age, body mass index (BMI), smoking status, socioeconomic status (SES) (Townsend quintile), prior 1 year cumulative GC dose at baseline, baseline Charlson comorbidity index [25], time-varying use of the DMARDs methotrexate, hydroxychloroquine, sulfasalazine and leflunomide and use of other DMARDs (penicillamine, azathioprine, cyclosporin, injectable gold) and time-varying use of non-steroidal anti-inflammatory drugs (NSAIDs) during follow-up. For a subgroup of the cohort who had the information available, the mean number of rheumatology outpatient visits per year and the mean number of GP visits per year was calculated and additionally adjusted for in a sensitivity analysis.

### Statistical analysis

Baseline characteristics were tabulated for the whole cohort, and stratified by ever use at the end of follow-up, to examine if there were any differences between ever users and never users.

Mortality rates with 95 % confidence intervals (CI) were estimated by dividing the number of deaths by the total number of person-years follow-up.

Primary analyses examined the association between GC exposure and time until death, using Cox proportional hazards regression [26], using the six GC exposure definitions described above. Associations between GC exposure and mortality (both all-cause and cause-specific) were estimated through crude, and fully adjusted hazard ratios (HR), with 95 % CI. The proportional hazards assumption was checked by testing the Schoenfeld residuals. The association between oral GC use and cause-specific mortality was further explored using the Fine and Gray competing risks approach [27]. All data analysis was performed using Stata/MP Version 12.1 (StataCorp, Texas).

### Missing data

The proportion of missing data for all confounders was assessed. If there was more than 5 % missing data the variable was included in a fully adjusted model 1, and assessed in a complete case analysis. Any variable that was significantly associated with the outcome or changed the hazard ratio for the primary exposure by at least 10 % was included in the analyses, and therefore imputed. Other variables were excluded from the analysis. If there was <5 % missing data the variable was included in the model, and only complete cases were included in the analyses.

### Exploring potential perimortal bias

Possible perimortal bias was explored in three ways. First, in order to explore whether GC therapy was being initiated in response to a terminal illness such as cancer, the distribution of cause-specific deaths in the first 6 months after GC initiation was compared to the distribution of cause-specific deaths more than 6 months after GC initiation in ever GC users. Second, the proportion of deaths was compared among two groups: (1) GC users who had oral GC therapy less than 6 months before death; and (2) GC users who had oral GC therapy more than 6 months before death but no GC use in the 6 months prior to death. Third, exposure during a 6 month period before death was excluded from the analyses to see if this had an impact on the results [28]. The same GC exposure models were used, although now based on the GC status at 6 months prior to death (see Figure A1 in Additional file 2).

The protocol for this study has been approved by Independent Scientific Advisory Committee for Medicines and Healthcare Regulatory Agency database research (Protocol number: 11_113RA4). As this study used routinely collected anonymised electronic health records consent was not required.

## Results

**Fig. 2:**
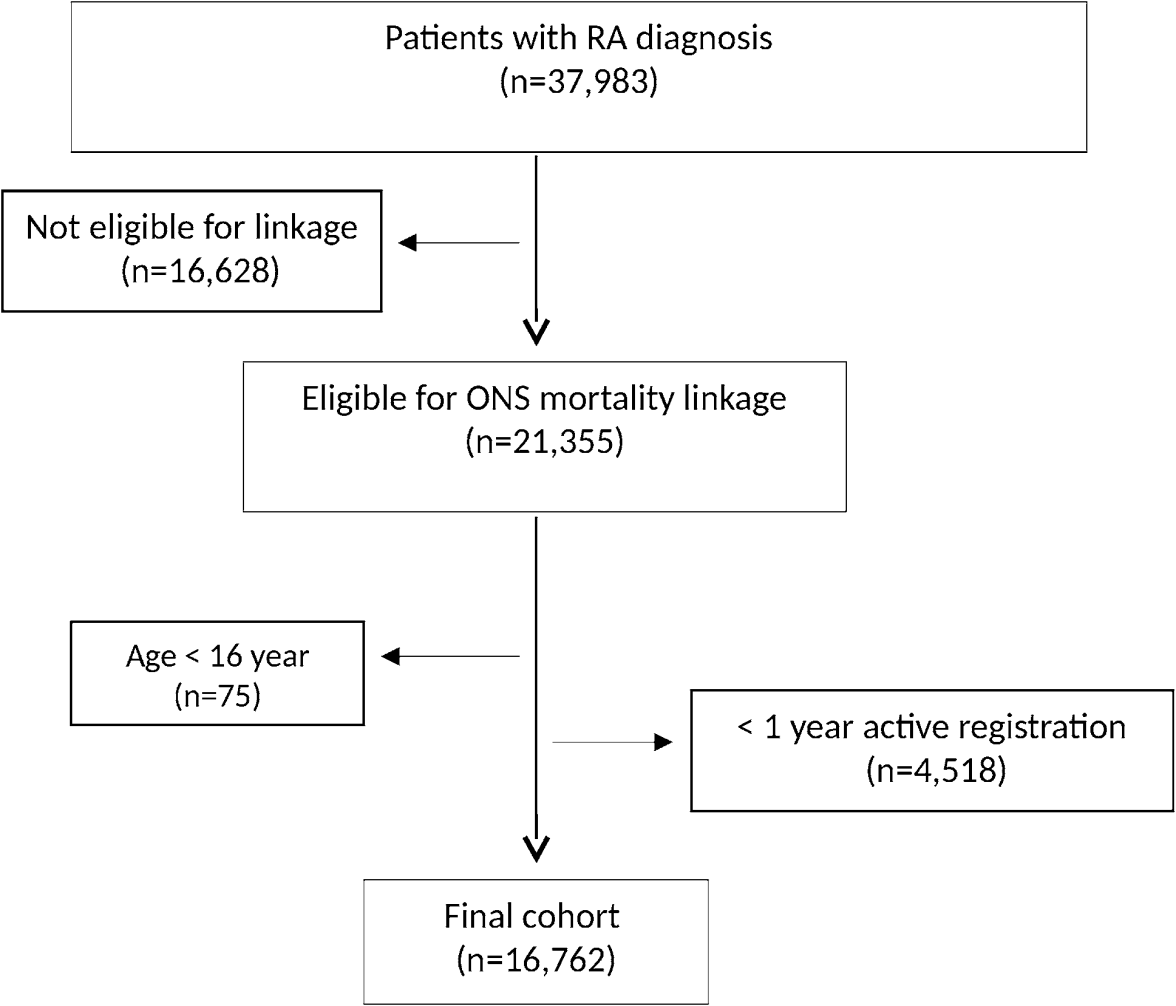
Flowchart of the ONS linked patient cohort

There were 37,983 patients identified with a diagnosis of RA, of whom 21,355 were eligible for ONS linkage. After applying the exclusion criteria, the cohort reduced to 16,762 patients (Fig. 2). Table 1 summarizes the patient characteristics of the whole cohort, ever GC users and non-users. 70 % of patients were female, with similar proportions in the GC and non-GC groups. Mean age at baseline was 60.2 years [standard deviation (SD): 14.6].

**Table 1:** Characteristics of the cohort, stratified by oral GC therapy status during follow-up

	All subjects	Never users	Ever users
Number of patients, n (%)	16,762	8395 (50.1)	8367 (49.9)
Follow-up time, total (person-years)	111,099	66,560	44,538
Females, n (%)	11,748 (70.1)	5945 (70.8)	5803 (69.4)
Age at baseline (years), mean (SD)	60.2 (14.6)	58.2 (14.9)	62.1 (14.1)
Body Mass Index at baseline			
Mean (SD)	26.8 (5.6)	26.9 (5.5)	26.8 (5.71)
Missing (%)	2763 (16.5)	1483 (17.7)	1280 (15.3)
Smoking status at baseline, n (%)			
Non smoker	7832 (46.7)	4115 (49.0)	3717 (44.4)
Ex smoker	3192 (19.0)	1489 (17.7)	1703 (20.4)
Current smoker	5227 (31.2)	2525 (30.1)	2702 (32.3)
Missing	511 (3.1)	266 (3.17)	245 (2.93)
Socioeconomic status quintile at baseline, n (%)			
First (least deprived)	3672 (21.9)	1871 (22.3)	1801 (21.5)
Second	4040 (24.1)	2031 (24.2)	2009 (24.5)
Third	3566 (21.3)	1746 (20.8)	1820 (21.8)
Fourth	3213 (19.2)	1601 (19.1)	1612 (19.3)
Fifth (most deprived)	2204 (13.2)	1112 (13.3)	1092 (13.1)
Missing	67 (0.4)	34 (0.4)	33 (0.4)
Prior history of GC use, n (%)	4138 (24.7)	484 (5.80)	3661 (43.8)
Charlson comorbidity index at baseline, mean (SD)	1.32 (0.70)	1.25 (0.64)	1.39 (0.76)
Methotroxate ever during follow-up, n (%)	8949 (53.4)	4020 (47.9)	4929 (58.9)
Hydroxycholoroquine ever during follow-up, n (%)	3728 (22.2)	1726 (20.6)	2002 (23.9)
Sulfasalazine ever during follow-up, n (%)	4793 (28.6)	2249 (26.8)	2544 (30.4)
Leflunomide ever during follow-up, n (%)	1465 (8.74)	455 (5.42)	1010 (12.1)
Other DMARDs ever during follow-up, n (%)^a^	4304 (25.7)	1683 (20.1)	2621 (31.3)

^a^Other DMARDS: penicillamine, azathioprine, cyclosporin, injectable gold

There were 8367 (50 %) patients who received at least one prescription for oral GCs. These patients were on average 4 years older, more likely to have received GCs in the 1 year prior to RA (44 vs. 6 %, respectively) and had higher DMARD use during follow-up compared to non-users. The mean baseline Charlson comorbidity index was slightly higher in ever users compared with never users (1.39 vs. 1.25) (Table 1).

During active GC prescriptions, the mean current daily dose (PED) was 7.5 mg (SD 6.9 mg). The mean cumulative dose (PED) among the 8367 patients who received GC therapy was 5.3 g (SD 6.0 g).

During a total of 111,099 person years, 2996 patients died (median follow-up of 6.1 years per person), giving an all-cause mortality rate of 27.0 deaths per 1000 person-years (pyrs) (95 % CI 26.0–28.0) (Table 2). In those never exposed to GCs the mortality rate was 15.5 deaths per 1000 pyrs, compared to 44.0 deaths per 1000 pyrs in those ever exposed to GCs.

**Table 2:** Underlying causes of death and crude mortality rates, overall and by ever GC use status

	All subjects		Never GC use^b^		Ever GC use	
	Events (%)	Mortality rate^a^	Events (%)	Mortality rate^a^	Events (%)	Mortality rate^a^
1 All-causes	2996	27.0 (26.0–28.0)	1034	15.5 (14.6–16.5)	1962	44.0 (42.1–46.0)
2 Cardiovascular diseases	1131 (100)	10.2 (9.60–10.8)	428 (100)	6.40 (5.84–7.07)	703 (100)	15.8 (14.7–17.0)
Ischemic heart diseases	581 (51.4)	5.23 (4.82–5.67)	207 (48.4)	3.11 (2.61–3.37)	374 (53.2)	8.39 (7.59–9.29)
Cerebrovascular diseases	247 (21.8)	2.22 (1.96–2.52)	121 (28.3)	1.82 (1.52–2.17)	126 (17.9)	2.83 (2.37–3.37)
Others	303 (26.8)	2.73 (2.44–3.05)	100 (23.3)	1.50 (1.24–1.83)	203 (28.9)	4.56 (3.97–5.23)
3 Neoplasms	639 (100)	5.75 (5.32–6.22)	191 (100)	2.87 (2.49–3.31)	448 (100)	10.1 (9.17–11.0)
Respiratory neoplasm	208 (32.6)	1.87 (1.63–2.14)	41 (21.5)	0.62 (0.45–0.84)	167 (37.3)	3.75 (3.22–4.36)
Digestive neoplasm	135 (21.1)	1.22 (1.03–1.44)	46 (24.1)	0.69 (0.52–0.92)	89 (19.9)	2.00 (1.62–2.46)
Others	296 (46.3)	2.66 (2.38–2.99)	104 (54.4)	1.56 (1.29–1.89)	192 (42.8)	4.31 (3.74–4.97)
4 Respiratory diseases	509 (100)	4.58 (4.20–5.00)	132 (100)	1.98 (1.67–2.35)	377 (100)	8.46 (7.65–9.36)
Respiratory infection	216 (42.4)	1.94 (1.70–2.22)	80 (60.6)	1.20 (0.97–1.50)	136 (36.1)	3.05 (2.58–3.61)
Lower respiratory diseases	205 (40.3)	1.85 (1.61–2.12)	32 (24.2)	0.48 (0.34–0.68)	173 (45.9)	3.88 (3.35–4.51)
Others	88 (17.3)	0.79 (0.64–0.98)	20 (15.2)	0.30 (0.19–0.47)	68 (18.0)	1.53 (1.20–1.94)
5 Others causes of death	717 (100)	6.45 (6.00–6.94)	283 (100)	4.25 (3.78–4.77)	434 (100)	9.74 (8.87–10.7)
Musculoskeletal diseases	201 (28.0)	1.81 (1.58–2.08)	67 (23.7)	1.00 (0.79–1.28)	134 (30.9)	3.01 (2.54–3.56)
Digestive diseases	158 (22.0)	1.42 (1.22–1.66)	68 (24.0)	1.02 (0.81–1.29)	90 (20.7)	1.64 (1.79–2.48)
Genitourinary diseases	75 (10.5)	0.68 (0.54–0.85)	22 (7.8)	0.33 (0.22–0.50)	53 (12.2)	1.19 (0.91–1.56)
Injury, poisoning and external causes	102 (14.2)	0.92 (0.76–1.11)	47 (16.6)	0.71 (0.53–0.93)	55 (12.7)	1.23 (0.95–1.61)
Others	181 (25.3)	1.63 (1.41–1.88)	79 (27.9)	1.19 (0.95–1.48)	102 (23.5)	2.29 (1.89–2.78)

^a^Mortality rates per 1000 patient-years

^b^Patients who had not yet used GCs could initially contribute person time to the ‘never GC use’ group, and then switch to ‘ever GC use’ person time on receipt of their first GC prescription

**Fig. 3:**
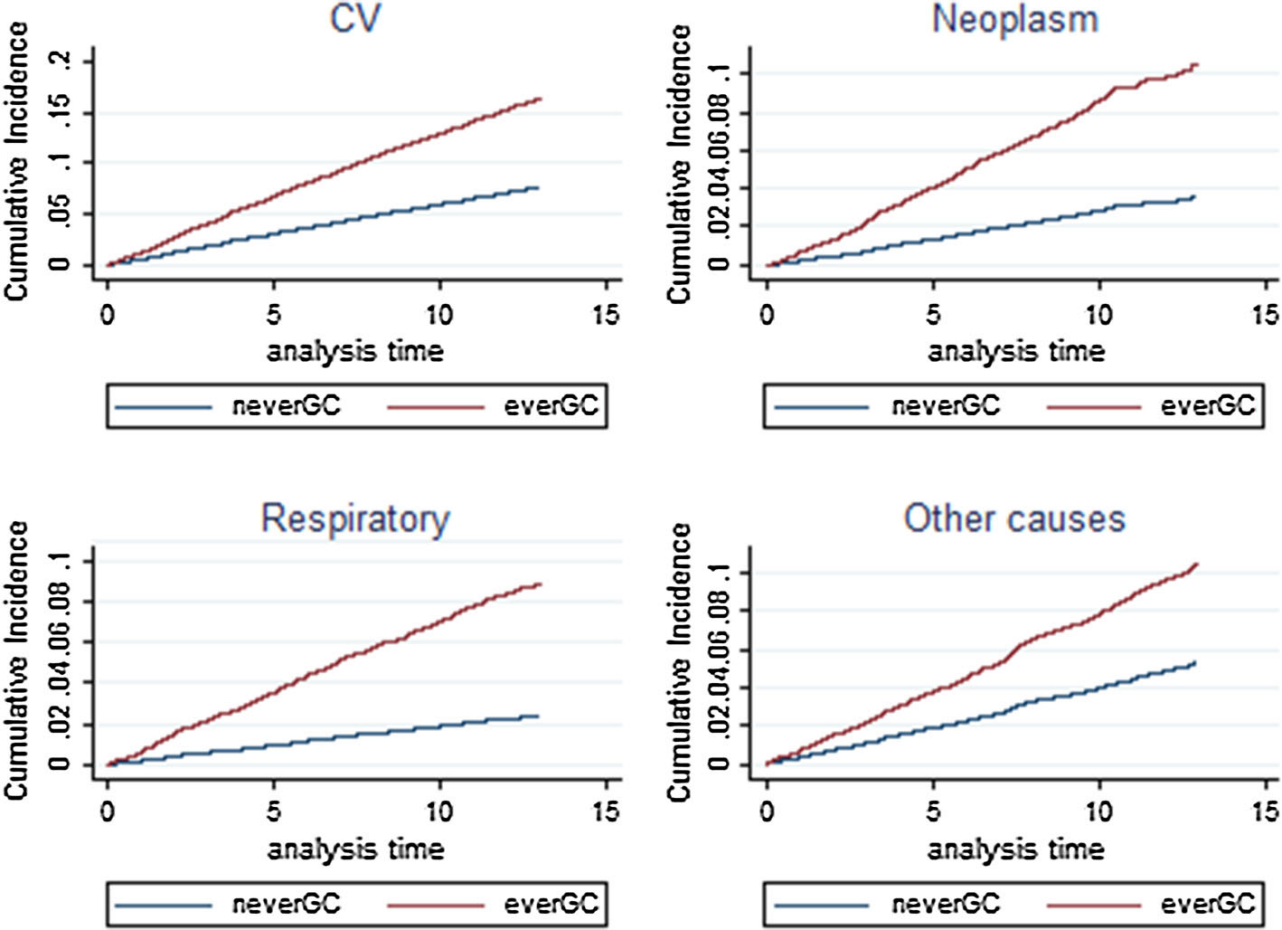
Cumulative incidence curves by GC status

Overall the most common cause of death was cardiovascular disease, followed by neoplasms and respiratory diseases. The underlying causes of death in the “other causes” category were mostly musculoskeletal (28.0 %). Figure 3 shows the cumulative incidence curves from Fine-Gray models [27] for the four categories of cause-specific mortality. Ever users had higher mortality rates in each cause-specific category compared to never users. For each category the mortality rate for ever users was higher than never users from the start of follow-up, and the mortality rate was consistent through follow-up for both exposed and unexposed groups.

Cardiovascular mortality rates were 15.8 deaths per 1000 pyrs in ever users compared to 6.4 deaths per 1000 pyrs in never users. Within this chapter, ischemic heart disease had the highest mortality rate for both ever and never users. Neoplasms had the second highest mortality rate for ever GC users. Conversely, the second highest mortality rate for never users was other causes of death. Respiratory diseases had the lowest mortality rate in both ever and never GC users (Table 2).

Table 3 shows the associations between oral GC use and risk of all-cause and cause-specific mortality, estimated through six alternative Cox models, adjusted for age, gender, smoking status, SES, prior cumulative dose of GC, baseline Charlson comorbidity index, time-varying NSAID use and time-varying DMARD use.

**Table 3:** Association between oral GC use and all-cause and cause-specific mortality (n = 16,187)

Model	Oral GC pattern	Adjusted hazard ratio (aHR) with 95 % CI^a^				
		All-cause mortality death = 2770	CV mortality death = 1039	Neoplasms death = 606	Respiratory diseases death = 468	Other causes death = 657
1	Ever use, (ref = never use)	1.97 (1.81–2.15)	1.66 (1.45–1.91)	3.20 (2.66–3.86)	2.64 (2.11–3.31)	1.39 (1.16–1.66)
2	Current use, (ref = non-use)	1.77 (1.62–1.93)	1.58 (1.37–1.83)	2.22 (1.84–2.68)	1.92 (1.57–2.36)	1.69 (1.41–2.02)
3	Current dose per 5 mg/day	1.33 (1.30–1.35)	1.21 (1.16–1.27)	1.46 (1.42–1.49)	1.36 (1.30–1.41)	1.25 (1.20–1.31)
4	Current dose category, (ref = non-use)					
	>0–4.9 mg	1.02 (0.87–1.20)	1.10 (0.85–1.41)	0.79 (0.51–1.22)	0.87 (0.57–1.33)	1.15 (0.85–1.57)
	5.0–7.4 mg	1.44 (1.26–1.64)	1.59 (1.31–1.94)	1.07 (0.75–1.52)	1.74 (1.30–2.32)	1.23 (0.93–1.63)
	7.5–14.9 mg	2.24 (1.98–2.54)	1.96 (1.59–2.42)	2.34 (1.75–3.13)	2.19 (1.62–2.97)	2.66 (2.09–3.38)
	15.0–24.9 mg	4.50 (3.61–5.62)	2.79 (1.80–4.31)	8.07 (5.41–12.0)	8.03 (5.31–12.2)	2.06 (1.09–3.90)
	≥25 mg	11.0 (8.87–13.6)	2.48 (1.23–4.99)	31.3 (23.5–41.9)	11.4 (6.84–19.0)	6.87 (4.01–11.8)
5	Cumulative dose since cohort entry (1000 mg/day)	1.06 (1.05–1.07)	1.05 (1.04–1.07)	1.06 (1.04–1.08)	1.07 (1.05–1.09)	1.07 (1.05–1.08)
6	Cumulative dose category (ref = non-use)					
	>0–959.9 mg	1.60 (1.42–1.81)	1.41 (1.16–1.72)	2.51 (1.97–3.21)	2.18 (1.61–2.95)	1.04 (0.79–1.36)
	960–3054.9 mg	1.83 (1.62–2.07)	1.38 (1.12–1.70)	3.84 (3.04–4.87)	2.24 (1.64–3.05)	1.16 (0.88–1.52)
	3055–7299.9 mg	2.11 (1.87–2.39)	1.91 (1.57–2.32)	3.31 (2.55–4.30)	2.65 (1.95–3.61)	1.48 (1.15–1.92)
	≥7300 mg	3.11 (2.74–3.52)	2.59 (2.11–3.18)	3.85 (2.90–5.10)	4.85 (3.59–6.55)	2.54 (1.98–3.25)

^a^Adjusted for gender, age, smoking status, SES, prior cumulative dose of GC, Charlson comorbidity index at baseline, time-varying NSAID use and time-varying DMARD use

BMI was the only potential confounder with higher than five percent of missing data (Table 1). When it was included in a complete case analysis of model 1 it did not alter the hazard ratio for GC use and was not significantly associated with mortality and so was not included in the fully adjusted models. Smoking and SES had <5 % missing data and were included in the fully adjusted models. All models consistently showed that risk of death was associated with GC use and increased with higher dosages of GCs. There was a nearly twofold greater risk of all-cause mortality in ever users, compared to never users (HR 1.97, 95 % CI 1.81–2.15). For cause-specific mortality, ever users had over a three times higher risk of death from neoplasms compared to never users (HR 3.20, 95 % CI 2.66–3.86). For both all-cause and cause-specific mortality, a similar pattern was seen for current use, though the point estimates were lower. For each 5 mg increase in GC dose there was a 33 % increased risk of all-cause mortality compared to non-users (HR 1.33, 95 % CI 1.30–1.35). Similar increased risks were seen for each of the cause specific mortality categories, with the highest risk seen for neoplasms (HR 1.46, 95 % CI 1.42–1.49).

The categorisation of current daily dose showed that for all-cause mortality, CV mortality and mortality due to respiratory diseases, a dose below 5 mg per day was not associated with an increased risk of death. Furthermore, for neoplasms and ‘other causes’, a dose of below 7.5 mg per day was not associated with an increased risk of death. However, as current daily dose increased above these doses, so too did the risk of death. Comparing between the hazard ratios for cause-specific mortality, the risk of cardiovascular mortality was notably lower than for the other three categories of death, for current GC dose above 7.5 mg.

There was a 6 % increased risk of all-cause mortality for each 1000 mg/day increase in cumulative dose since cohort entry (HR 1.06, 95 % CI 1.05–1.07). Similar increases in risk were seen for each cause-specific mortality category. Categorisation of cumulative dose showed a dose response increased risk of all-cause mortality in each category of cumulative dose, with risk of death increasing with increased categories of cumulative dose. The exception to this was for other causes of death, where there was not an increased risk of death from other causes with cumulative doses up to 3054.9 mg (Table 3). Additional adjustment for mean number of rheumatology outpatient visits per year and mean number of GP visits per year in general increased the risk of all-cause mortality and cause-specific mortality, but did not alter the significance, except for the lowest current dose category (0-4.9 mg) where a significantly reduced risk of mortality due to neoplasms was seen (Online resource Table A1).

### Perimortal bias

The mortality rate in the first 6 months following GC therapy initiation was 56.5 deaths per 1000 pyrs, compared to 42.8 deaths per 1000 pyrs beyond 6 months after GC initiation. The rate of neoplasm deaths was higher in patients in the first 6 months following GC initiation (23.5 per 1000 pyrs compared to 8.7 per 1000 pyrs beyond 6 months) (Online Resource Table A2).

Of those who died (N = 2996), 1962 patients ever used GCs. Of these, 1576 patients used GCs during the 6 months prior to death and 368 last used GCs more than 6 months prior to death. Those who used GC in the 6 months prior to death had a higher proportion of deaths due to respiratory, neoplasms and other causes, but a lower proportion of CV deaths, compared to those patients who received GC therapy more than 6 months prior to death. For example 23.4 % of those who used GCs during the 6 months prior to death died from neoplasms, compared to 20.7 % in those who used GCs more than 6 months prior to death (Online Resource Table A3).

After the exclusion of GC information in the 6 months prior to death, the association between ever use and all-cause mortality was reduced but remained statistically significant (HR 1.64, 95 % CI 1.50–1.79). A similar reduction in hazard ratio was seen for cause-specific mortality, in particular neoplasm mortality where ever users had only a 76 % increased risk of death from neoplasms (HR 1.76, 95 % CI 1.47–2.10), compared to a threefold greater risk when the 6 months prior to death was included (HR 3.20, 95 % CI 2.66–3.86). In Model 4, the magnitude of risk was reduced for the highest dose category of >25 mg PED for all-cause and each cause-specific mortality. Excluding the exposure data from 6 months prior to death had the biggest impact on deaths caused by neoplasms, with hazard ratios falling from 8.07 (95 % CI 5.41–12.0) to 3.42 (95 % CI 1.87–6.28) for 15–25 mg, and from 31.3 (95 % CI 23.5–41.9) to 5.66 (95 % CI 2.80–11.4) for >25 mg. Full results for models 1-6 following exclusion of GC information in the 6 months prior to detail are shown in Online Resource Table A4.

### Unmeasured confounding

The cause-specific analyses found an association between oral GC use and death from other causes, supporting the possibility of unmeasured confounding. To explore this, a post hoc sensitivity analysis was conducted using the rule out approach [29, 30]. This approach finds the minimum effect an unmeasured confounder would need to have to remove statistical significance. It was found that an unmeasured confounding factor with 40 % prevalence would have to increase the relative risk of mortality by a factor 3 and at the same time increase the odds of GC exposure by a factor of 3.5 in order to fully remove the association found between ever use and mortality risk due to other causes (HR 1.39, 95 % CI 1.16–1.66). For each of the other causes of death the unmeasured confounders would need to increase the relative risk of mortality and the odds of GC exposure by too large an amount for them to explain the result fully. For example, an unmeasured confounding factor for CV mortality would have to increase the relative risk of CV mortality by a factor of 3 and increase the odds of GC exposure by a factor of 7.7 in order to remove the association found, which seems unlikely. Similarly, an unmeasured confounder with increased risk of death by a factor below 3 cannot plausibly explain the observed association between GC exposure and CV mortality.

## Discussion

This study examined the association between oral GC therapy and mortality rates in a cohort of patients with RA in the UK. Ever GC use and current GC use was associated with an increased risk of all-cause mortality and cause-specific mortality, with a largely consistent dose–response effect. An increase in current dose of 5 mg per day was associated with an increased risk of death, however categorisation showed that taking <5 mg per day at the time of death did not increase the risk of all-cause mortality or cause-specific mortality, and taking <7.5 mg per day at the time of death did not increase this risk of death from neoplasms or other non-CV and non-respiratory causes. In addition, moderate to high doses of GC therapy were associated with a lesser risk of CV deaths compared to neoplasm, respiratory and other causes of death, which might suggest GC therapy has a less harmful effect on CV mortality.

The study showed that perimortal bias partially explained some of the results, especially at higher doses. Perimortal bias is important to consider for a number of reasons. GCs can be used to treat diseases that might develop through the course of follow-up, and where that disease is the leading cause of death. For example, if a patient were to develop a malignancy, they might start GC therapy as part of their cancer treatment which would lead to a positive association between GCs and (cancer-related) mortality. Similarly, end of life care might lead to a switch from disease-modifying anti-rheumatic drug (DMARD) therapy (that requires regular blood monitoring) to GC therapy, again generating an association between GC use and death.

When GC use in the 6 months prior to death was removed, the association between ever GC use and all-cause mortality remained significant, but the risks were reduced. This was mainly influenced by the large reduction in risk of death from neoplasms, where there is a clear possibility of perimortal bias: GCs are prescribed as a treatment for cancer [31]. Initial signals of possible perimortal bias were evident in the magnitude of the association between high-dose GCs and risk of death due to neoplasm (HR 31.3, 95 %CI 23.5–41.9).

The all-cause mortality rate for this study was 27 deaths per 1000 person-years, and the cardiovascular mortality rate was 10 deaths per 1000 person-years. This was higher than a recent cohort study in the UK (Norfolk Arthritis Registry (NOAR)) [32] where rates were 20–21 per 1000 person-years and 7–8 per 1000 person years for all-cause mortality and cardiovascular mortality respectively. This would be expected as NOAR includes patients with early inflammatory arthritis, whereas this study included patients with a higher baseline age and with RA only, and therefore more severe disease.

Our findings are in agreement with some previous studies [5, 12–15, 17] which have investigated all-cause mortality or CV mortality in association with GC use. Caplan et al. [5] found an increased risk of death with current GC use, with an adjusted odds ratio of 2.2 (95 % CI 1.9–2.7) and an increased risk of death with increasing duration of GC treatment. del Rincon et al. [12] found a GC dose-dependent increase in death from all causes (HR 1.07 per 1 mg/day (95 % CI 1.05–1.08) and CV cause with a similar point estimate. They also showed that there was a dose response association for cumulative dose for all-cause and CV mortality with a threshold of 40 g. Listing et al. [13] showed that GC doses higher than 5.0 mg per day were significantly associated with increased all-cause mortality, independent of disease activity. Treatment with prednisolone higher than 15 mg per day was associated with 3.4-fold (95 % CI 2.01–5.86) increased risk of all-cause mortality compared with non-use. Our findings of probable perimortal bias, however, might suggest that the hazard ratios reported in these previous studies are over-estimates of the true effect.

An important finding of this study was the absence of an association between both all-cause and cause-specific mortality and GC doses lower than 5 mg per day. This may reflect either a low risk of adverse events at this dose, or at least a favourable balance between the harms and the biologically plausible benefits through their anti-inflammatory effects [33]. This finding replicates similar findings from Listing et al. [13] and del Rincon et al. [12], which showed that doses lower than 5 and 8 mg PED, respectively, had no association with mortality risk.

The strengths of this study are firstly its size, with nearly 3000 deaths in 16,762 patients. This meant the study had greater power to detect differences in mortality rates and allowed us to explore cause-specific mortality. We were thus able to see an increased rate of respiratory deaths, accepting the possibility of perimortal bias but also likely driven by a causal increased risk of respiratory infection [9]. Second, the study used linkage to the national mortality register, providing robust and complete information on cause of death for all patients in the study. Third, time-varying covariates for DMARDs, NSAIDs and GCs, were used to allow more accurate estimation compared to time-independent variables for these drugs. Fourth, a range of patterns of GC use were explored including GC use, GC daily dose, and cumulative dose since cohort entry and their categorical variables compared with non GC use in association with risk of death. This approach allowed some consideration of the impact of dose, duration and timing of treatment on mortality risk. For example, the finding that the highest current dose category was associated with very high HRs for neoplasm, respiratory and other causes of death, whilst the highest quartile had notably lower HRs, suggests that high doses may be used at the end of life when cumulative exposure is less of an issue. We also explored possible perimortal bias which has not been considered in previous studies. Moreover, we examined oral GC therapy in association with cause-specific mortality beyond CV mortality which has not been investigated in previous studies.

There were some limitations with the study. The prescription data from the CPRD dataset are reliable in terms of drugs prescribed, but does not cover drugs prescribed in secondary care only, such as biologic DMARDs, or over the counter use of NSAIDs; although it has been shown that biologic DMARDs are not associated with an increased mortality compared to standard DMARDs [34]. In addition there may have been some exposure misclassification because of assumptions in data preparation, missing data, patient adherence, injectable steroids and hospital administered GC, although the latter is likely to be minimal as UK rheumatologists typically make recommendations for oral GC treatment to GPs. Like all observational studies, the impact of confounding and bias needed consideration. A range of possible confounders were adjusted for, including time-varying exposure to DMARDs and NSAIDS, and healthcare utilisation variables as surrogate measures of RA disease severity. Although we didn’t have direct measures of disease severity, previous studies that did adjust for clinician-reported disease severity found a persistent association between GC use and mortality [13]. It is thus likely that there was some residual confounding by disease severity. In terms of possible residual or unmeasured confounders affecting the results, of which disease severity is one, sensitivity analyses showed that these would need to be very large to fully explain the results. So even though, for example, high cumulative disease severity has been shown to be associated with lymphoma [35] this would not fully explain the results seen. Adjusting for the Charlson comorbidity index at baseline was expected to control for key comorbidities that contribute to an increased risk of mortality. The main difference at baseline between GC users and non-GC users was prior GC use, which was much higher in GC users (44 vs. 6 %). It may have been that this group was particularly susceptible to death, and any association between GC use and death may have been exaggerated. However, prior GC use was adjusted for so the results should not be biased.

It is very challenging to understand the true causal relationship between oral GC use and mortality from an observational study due to the complex relationships between the indication for treatment (that changes through time) and the outcome, as well as the granularity of the data from a population necessarily large to support the analysis. Nonetheless, despite this blurring of causality by bias and confounding, some important messages emerge. Doses <5 mg were not associated with an increased risk of death. This absent risk is not explained by confounding by disease severity (where you would expect mortality to be higher in the treated compared to non-treated), or by perimortal bias where you would again expect an increased risk compared to non-use. The lower dose-specific hazard ratios for cardiovascular deaths compared to the hazard ratios seen for the other causes of death raises the interesting hypothesis that GC therapy might have a beneficial effect on cardiovascular mortality; yet a protective effect is impossible to conclude with certainty as there is a statistically significant increased risk for CV mortality with all doses above 5 mg. Disentangling these complex factors is impossible, but the large population observational research raises questions that can feed back into more targeted studies, both basic science and epidemiological.

## Conclusions

This study has found that GC use is associated with an increased risk of death in RA, both all-cause and cause-specific mortality, which is partially explained by perimortal bias. Importantly, doses of below 5 mg PED were not associated with an increased risk of death. There is a suggestion that GCs may have a less harmful effect on CV mortality compared to their association with other cause-specific mortality, but targeted research is required to examine this signal further.

## Electronic supplementary material

Below is the link to the electronic supplementary material.

Supplementary material 1 (PDF 432 kb)
