# Supplementary material for: Oral glucocorticoid therapy and all-cause and cause-specific mortality in patients with rheumatoid arthritis: a retrospective cohort study
Source: Eur J Epidemiol. 2016 Jun 2;31(10):1045–55. doi: 10.1007/s10654-016-0167-1 (PMC5065607; doi:10.1007/s10654-016-0167-1)
Supplement: Supplementary file 1 — Supplementary material 1 (PDF 432 kb) [file 10654_2016_167_MOESM1_ESM.pdf]

Online supplements

A1. Algorithm for definition of GC dose and duration

- Prescription duration was identified using the following approaches:

  (1) Number of days the prescription was for;

  (2) If this was not available, calculating the number of days by dividing the total amount of oral GC prescribed by the number per day for that period,

  (3) If total amount of GC prescribed or number per day was missing, the mean duration for subsequent prescriptions for that patient (if there was a subsequent GC prescription for the patient) was used, or

  (4) The mean prescription duration for all individuals using the same drug (if there was no subsequent GC prescription for the patient).

- If there was an overlap between two prescriptions, it was assumed the patients started the new prescription after finishing their first prescription. The maximum period of current exposure was set at one year for continuous prescription of oral GC.

- The daily dose of oral GC was calculated by multiplying the number of tablets prescribed per day by the strength of that particular oral GC.

Figure A1. Removing 6 months period before death for checking the impact of perimortal bias

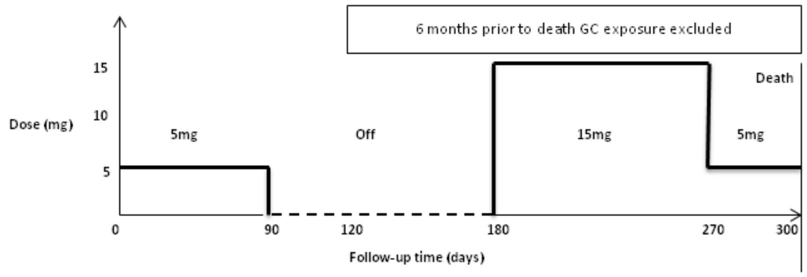

GC exposure type	0-90 days	90-120 days	120-300 days (GC exposure for 6 months prior death was excluded and the last exposure status from 6 months earlier was carried forward)
Ever GC status	Yes	Yes	yes
Current status	Yes	No	No
Current GC dose (mg)	5	0	0
Current dose, category (mg)	5-7.4	0	0
Cumulative dose at end of each episode (mg)	450	450	450
Cumulative dose category (mg)	0-959.9	0-959.9	0-959.9

Table A1. Association between oral GC use and mortality overall and by cause-specific with additional adjustment (N=11523).

Model	Oral GC pattern	Adjusted hazard ratio (aHR) with 95% CI *				
		All-cause mortality death=1357	CV mortality death=483	Neoplasms death=639	Respiratory diseases death=243	Other causes death=320
1	Ever use, (ref=never use)	2.26 (1.98-2.58)	2.00 (1.61-2.48)	3.62 (2.73-4.81)	2.90 (2.07-4.07)	1.50 (1.15-1.95)
2	Current use, (ref=non-use)	1.83 (1.62-2.06)	1.69 (1.38-2.06)	2.20 (1.71-2.84)	1.84 (1.40-2.43)	1.76 (1.38-2.24)
3	Current dose per 5mg /day	1.35 (1.32-1.38)	1.23 (1.15-1.31)	1.47 (1.42-1.52)	1.35 (1.28-1.43)	1.29 (1.22-1.36)
4	Current dose category, (ref=non-use)					
	>0-4.9mg	0.95 (0.76-1.20)	1.09 (0.77-1.56)	0.45 (0.21-0.97)	0.73 (0.40-1.32)	1.23 (0.82-1.85)
	5.0-7.4mg	1.53 (1.29-1.83)	1.80 (1.37-2.35)	1.07 (0.67-1.71)	1.84 (1.27-2.68)	1.22 (0.83-1.78)
	7.5-14.9mg	2.26 (1.90-2.68)	2.05 (1.53-2.75)	2.29 (1.55-3.40)	1.94 (1.28-2.94)	2.77 (2.01-3.83)
	15.0-24.9mg	5.74 (4.30-7.66)	2.68 (1.37-5.24)	12.4 (7.77-19.7)	9.10 (5.26-15.7)	2.78 (1.22-6.33)
	≥25mg	11.6 (8.70-15.5)	3.16 (1.30-7.68)	35.3 (23.7-52.5)	8.94 (4.14-19.3)	7.54 (3.68-15.4)
5	Cumulative dose since cohort entry (1000mg/day)	1.06 (1.05-1.07)	1.05 (1.03-1.07)	1.06 (1.04-1.08)	1.07 (1.05-1.09)	1.06 (1.04-1.08)
6	Cumulative dose category (ref=non-use)					
	>0-959.9mg	1.80 (1.50-2.16)	1.78 (1.33-2.39)	2.66 (1.84-3.85)	2.17 (1.36-3.45)	1.11 (0.74-1.66)
	960-3054.9mg	2.08 (1.73-2.49)	1.63 (1.19-2.22)	4.22 (2.98-5.98)	2.37 (1.50-3.75)	1.29 (0.88-1.90)
	3055-7299.9mg	2.28 (1.91-2.72)	2.08 (1.55-2.80)	3.84 (2.65-5.55)	2.75 (1.78-4.26)	1.47 (1.01-2.12)
	≥7300mg	3.39 (2.86-4.03)	2.91 (2.18-3.87)	4.64 (3.17-6.77)	5.17 (3.44-7.78)	2.39 (1.70-3.35)

*Adjusted for gender, age, smoking status, SES, prior cumulative dose of GC, Charlson comorbidity index at baseline, time-varying NSAID use, time-varying DMARD use, mean number of rheumatology visits per year and mean number of GC visits per year.

Table A2. Mortality rate by cause of death following GC therapy initiation in ever GC users

	Within the six months	Time period after the first six months
**All-cause**		
Events	228	1,734
Time at risk (pyrs)	4,039	40,499
Rate per 1000pyrs	56.5 (49.6-64.3)	42.8 (40.8-44.9)
**CV**		
Events	58	645
Time at risk (pyrs)	4,039	40,499
Rate per 1000pyrs	14.4 (11.1-18.6)	15.9 (14.7-17.2)
**Neoplasm**		
Events	95	355
Time at risk (pyrs)	4,039	40,499
Rate per 1000pyrs	23.5 (19.2-28.7)	8.72 (7.85-9.67)
**Respiratory**		
Events	32	345
Time at risk (pyrs)	4,039	40,499
Rate per 1000pyrs	7.92 (5.60-11.2)	8.52 (7.67-9.47)
**Others**		
Events	43	391
Time at risk (pyrs)	4,039	40,499
Rate per 1000pyrs	10.6 (7.89-14.4)	9.65 (8.74-10.7)

Table A3. The proportion of cause of death by GC use status

	Never GC use N (%)	GC used in < 6 months before death N (%)	GC used in ≥ 6 months before death N (%)	Total	
CV	428 (41.4)	540 (34.2)	163 (42.2)	1,131 (37.8)	
Neoplasm	191 (18.5)	368 (23.4)	80 (20.7)	639 (21.3)	
Respiratory	132 (12.8)	313 (19.9)	64 (16.6)	509 (17.0)	
Others	283 (27.4)	355 (22.5)	79 (20.5)	717 (23.9)	
Total	1,034 (100)	1,576 (100)	386 (100)	2,996	Pearson chi2(6)=44.7 p- value (<0.001)

Table A4. Association between selected oral GC models and all-cause mortality and cause-specific mortality after removal of GC information for the 6 months prior to death

	Adjusted hazard ratio (aHR) with 95% CI *				
**Oral GC pattern**	All-cause (death=2,666)	CV (death=990)	Neoplasm (death=589)	Respiratory (death=453)	Others (death=634)
Ever use, (ref=never use)	1.64 (1.50-1.79)	1.57 (1.36-1.81)	1.76 (1.47-2.10)	2.37 (1.90-2.97)	1.25 (1.04-1.50)
GC use in 6 months prior death, (ref=non-use)	1.82 (1.66-1.99)	1.54 (1.32-1.78)	1.98 (1.63-2.40)	2.03 (1.65-2.50)	2.00 (1.67-2.39)
GC dose in 6 months prior death (5mg /day)	1.30 (1.26-1.33)	1.23 (1.17-1.29)	1.32 (1.25-1.40)	1.35 (1.28-1.43)	1.33 (1.26-1.40)
GC dose category in 6 months prior death, (ref=non-use)					
>0-4.9mg	1.32 (1.14-1.53)	1.12 (0.87-1.44)	1.56 (1.14-2.15)	1.22 (0.85-1.77)	1.51 (1.13-2.02)
5.0-7.4mg	1.52 (1.34-1.74)	1.40 (1.13-1.73)	1.74 (1.30-2.31)	1.64 (1.21-2.22)	1.46 (1.11-1.92)
7.5-14.9mg	2.56 (2.26-2.89)	2.09 (1.69-2.58)	2.37 (1.78-3.18)	2.99 (2.26-3.96)	3.18 (2.51-4.03)
15.0-24.9mg	3.68 (2.85-4.75)	3.34 (2.19-5.08)	3.42 (1.87-6.28)	5.12 (3.02-8.68)	3.20 (1.81-5.64)
≥25mg	4.15 (2.84-6.08)	1.15 (0.37-3.59)	5.66 (2.80-11.4)	8.28 (4.23-16.2)	4.93 (2.33-10.5)
Cumulative dose since cohort entry (1000mg/day)	1.06 (1.05-1.07)	1.05 (1.04-1.07)	1.05 (1.03-1.07)	1.07 (1.05-1.09)	1.07 (1.05-1.08)
Cumulative dose category (ref=non-use)					
>0-959.9mg	1.32 (1.17-1.50)	1.26 (1.02-1.55)	1.52 (1.19-1.94)	1.90 (1.40-2.58)	0.94 (0.72-1.24)
960-3054.9mg	1.55 (1.37-1.76)	1.47 (1.20-1.81)	1.75 (1.35-2.27)	2.41 (1.80-3.23)	1.05 (0.79-1.38)
3055-7298.9mg	1.73 (1.53-1.97)	1.78 (1.45-2.18)	1.92 (1.47-2.51)	2.11 (1.53-2.91)	1.33 (1.02-1.74)
≥7299mg	2.59 (2.28-2.95)	2.33 (1.87-2.89)	2.31 (1.73-3.08)	4.22 (3.10-5.75)	2.30 (1.78-2.97)

* Adjusted for gender, age, smoking status, SES, prior cumulative dose of GC, Charlson comorbidity index at baseline, time-varying NSAID use and time-varying DMARD use
